# Insight into synergetic mechanisms of tetracycline and the selective serotonin reuptake inhibitor, sertraline, in a tetracycline-resistant strain of *Escherichia coli*

**DOI:** 10.1038/ja.2017.78

**Published:** 2017-07-12

**Authors:** Lili Li, Sofie Kromann, John Elmerdahl Olsen, Søren Wedel Svenningsen, Rikke Heidemann Olsen

**Affiliations:** 1 College of Light Industry and Food Sciences, South China University of Technology, Frederiksberg C, Denmark; 2 Department of Veterinary and Animal Sciences, Faculty of Health and Medical Sciences, University of Copenhagen, Frederiksberg C, Denmark; 3 Department of Chemistry Animal University of Copenhagen, Frederiksberg C, Denmark

## Abstract

Sertraline, an antidepressive drug, has been reported to inhibit general bacterial efflux pumps. In the present study, we report for the first time a synergistic effect of sertraline and tetracycline in a TetA-encoded tetracycline-resistant strain of *Escherichia coli*. Synergy between sertraline and tetracycline in an *E. coli* strain with TetA-mediated tetracycline resistance (*E. coli* APEC_O2) was assessed by the MIC and checkerboard assays. The global transcriptome of *E. coli* APEC_O2 exposed to ½ MIC concentrations of sertraline and/or tetracycline was analyzed to elucidate the interaction mechanism between sertraline and tetracycline. The fractional inhibitory concentration index for tetracycline and sertraline in *E. coli* APEC_O2 was 0.5. In addition, in the presence of ½ MIC of sertraline, the sensitivity of *E. coli* APEC_O2 to tetracycline could be restored according to clinical standards (from 64 to 4 mg l^−1^). RNA data suggest changes in respiration that is likely to decrease intracellular pH and thereby the proton-motive force, which provides the energy for the tetracycline efflux pump. Furthermore, sertraline and tetracycline may induce a change from oxidation to fermentation in the *E*.*coli*, which further decreases pH, resulting in cell death. This study shows that sertraline interacts with tetracycline in a synergistic and AcrAB-TolC pump-independent manner. The combinational treatment was further shown to induce many changes in the global transcriptome, including altered *tet*A and *tet*R expression. The results indicate that sertraline may be used as a helper compound with the aim to reverse tetracycline resistance encoded by *tetA*.

## Introduction

Several neuroleptic drugs; for example, phenothiazine and selective serotonin reuptake inhibitors, have been reported to have anti-microbial properties in high concentration, while having anti-microbial ‘helper-compound’ properties in lower concentration.^1, 2, 3, 4^ For the latter group, evidence suggests that the effect is due to inhibition of broad-specificity efflux pumps.^5^ These efflux pumps recognize a spectrum of noxious agents, including antibiotics such as tetracycline, and extrude these from the bacterial cytoplasm.^6^ Selective serotonin reuptake inhibitor compounds may also produce an anti-microbial response by themselves, a mechanism that is only poorly understood and investigated. In *E*. *coli*, the tripartate AcrAB-TolC system is the best-studied efflux pump system. Sertraline, a selective serotonin reuptake inhibitor compound, has been shown to decrease the MIC of tetracycline for strains that overproduce the AcrAB-TolC pump system.^7^

In addition, antibiotic-specific efflux pumps; for example, the TetA pump, which is a proton-motive force-dependent tetracycline-specific pump,^8^ is frequently observed in highly antibiotic-resistant bacteria. TetA pumps are commonly found in *E. coli* strain with clinically relevant tetracycline resistance,^9^ but how sertraline impact *tetA*-encoded resistance remains to be investigated.

The aim of the present study was to investigate the effect of combined sertraline and tetracycline exposure to evaluate the ‘helper-compound’ properties of sertraline against *tetA*-encoded tetracycline resistance in *E. coli*. To elucidate the mechanism behind the synergy observed between sertraline and tetracycline, the global transcriptomic response of a *tetA*-encoded tetracycline-resistant *E. coli* was further characterized.

## Materials and methods

### Bacterial strain characteristics and susceptibility testing

The MIC for sertraline hydrochloride and tetracycline (Sigma, Copenhagen, Denmark) were determined for a collection of 84 *E*.*coli* isolates. The strains originated from a random collection of porcine commensal *E*.*coli* strains from the strain collection of Department of Veterinary and Animal Sciences, UCPH. The MIC of some of the strains have previously been reported,^10^ but the MIC determination was repeated in the current investigation.

The tetracycline-resistant strain, *E. coli* APEC_O2, was chosen for detailed characterization of the response of tetracycline-resistant *E. coli* to sertraline, tetracycline or a combination hereof. The isolate originates from a diseased chicken.^11^ It possesses a resistance plasmid encoding resistance towards eight different anti-microbials, including tetracycline.^12^ In *E. coli* APEC_O2, tetracycline resistance is mediated by a tetracycline-specific efflux pump, TetA.^13^

The MIC determination was carried out following CLSI guidelines.^14^ Mueller–Hinton (MH) broth (Sigma) was supplemented with tetracycline or sertraline and distributed in individual microtiter plates with twofold dilution increase, in concentrations of the compounds ranging from 0 to 1024 mg l^−1^ and 0 to 128 mg l^−1^ for tetracycline and sertraline, respectively. pH of the MH broth was measured for MH broth unsupplied and supplied with the maximum concentration of each compound. *E*.*coli* in saline suspensions were prepared from overnight cultures on blood agar and adjusted to a 0.5 McFarland turbidity standard. The suspensions were diluted 1:100 in MH and this suspension was used as inoculum of the wells, giving a final concentration of ~5 × 10^5^ CFU ml^−1^. After twice determination of MIC for tetracycline and sertraline for each strain, MIC of tetracycline was subsequently determined twice in broth supplemented with ½ MIC of sertraline, otherwise following the description above. The inoculated microtiter plates were incubated aerobically at 37 °C for 18–22 h.

For sertraline, the MBC was determined by plating 100 μl from wells where no growth was observed onto MH agar plates. The plates were incubated at 37 °C for 18–20 h before growth was determined. The MBC was classified as the concentration where ⩾99% reduction in bacterial cell count was observed compared with CFU of 100 μl untreated culture of the same strain.

MIC for tetracycline for *E*.*coli* APEC_O2 cocultured with known inhibitors of general efflux pumps (Phe-Arg β-naphthylamide, chlorpromazine and thioridazine (Sigma), respectively) was determined according to the method stated above.

The MIC of penicillin, kanamycin and erythromycin (all compounds obtained from Sigma), with and without supplementation with ½ MIC of sertraline, were determined for strain APEC_O2 as well.

### Growth conditions

Growth experiments were performed in triplicate on a BioScreen C (Oy Growth Curves Ab, Helsink, Finland) for 24 h at 37 °C. A volume of 200 ml of MH broth was inoculated with a culture of *E. coli* APEC_O2 growing overnight to a final cell density of 10^6^ CFU ml^−1^. The concentration of the culture was adjusted using a Sensititer Nephelometer (Thermo Scientific TM, Roskilde, Denmark) with a 0.5 McFarland standard (1–2 × 10^8 ^CFU ml^−1^). The cultures were supplemented with sertraline and tetracycline alone or in combination. An untreated control was included. The OD (recorded with a 600 nm filter) was measured every 5 min with continuous shaking.

Time-kill assays (triplicates) were used to determine the rate of bacterial killing when exposed to sertraline and/or tetracycline following a previously described protocol.^15^ *E. coli* APEC_O2 was grown to early exponential phase and treated with concentrations equivalent to either ½ MIC of sertraline, ½ MIC of tetracycline or ½ MIC of sertraline combined with ½ MIC of tetracycline in MH broth. Growth was monitored by OD_600_ measurements on a Helios spectrophotometer (Thermo Electron Corporation Instrument, Beverly, MA, USA) and by CFU ml^−1^ determinations by plating 10-fold serial dilutions on MH agar plates.

### Checkerboard assays

Synergistic effect of sertraline on tetracycline activity against *E. coli* APEC_O2 was evaluated by checkerboard method with 96-well microtiter plates using MH broth, as described elsewhere.^16^ For each combination, the fractional inhibitory concentration (FIC) was calculated as the MIC of the tetracycline in combination sertraline divided by the MIC of the tetracycline alone and likewise for sertraline. The FIC indexes were derived from summation of individual FICs.^17^

### Isolation of total cellular RNA

RNA was isolated from APEC_O2 exposed to three different treatments (sertraline (16 mg l^−1^), tetracycline (32 mg l^−1^) or a combined treatment of sertraline (8 mg l^−1^) and tetracycline (4 mg l^−1^) in addition to an untreated control. The concentrations corresponded to ½ MIC for individual tetracycline or sertraline treatment, and to ½ MIC for tetracycline/sertraline combined treatment. For each growth condition, three colonies of APEC_O2 cultured at 37 °C in MH broth unsupplemented or supplemented with sertraline, tetracycline or sertraline/tetracycline (ST) in the concentrations stated above, allowing bacteria to reach the early logarithmic growth phase (OD_600_=0.2–0.4). RNA from each of three replicates of each growth conditions were extracted using the RiboPure Bacteria Kit (Thermo Fisher Scientific) according to the manufacturer's instructions. RNA extraction samples were subjected to DNAse treatment and cleanup using the RNeasy Kit (Qiagen, Hilden, Germany). RNA integrity was confirmed using a TapeStation with RNA High-Sensitivity Screen Tapes (Agilent).

### Library preparation and sequencing

rRNA was removed by the RiboPure Kit (Illumina, Little Chesterford, UK). Transcriptome libraries were prepared using the stranded TruSeqmRNAseq protocol, which enables strand-specific identification of transcripts. The 12 samples were pooled in equimolar concentrations and sequenced on an IlluminaMiSeq using the 1 × 150 bp MiSeq Reagent Kit v.3 (Illumina).

### Bioinformatic processing and analysis

Raw sequence reads in fastq format were trimmed using Trimmomatic v.0.36 with the settings; LEADING:3TRAILING:3 SLIDINGWINDOW:4:15 MINLEN:50 and removing Illumina adapters if found.^18^ The trimmed transcriptome reads were mapped to features annotated as CDS, rRNA or tRNA in the annotated genome of *E*.*coli* APEC_O2 and the two associated plasmids pAPEC-O2-CoIV and pAPEC-O2-R, using bowtie-2 as default parameters.^19^ For each mapping the number of reads mapping to a specific gene was calculated using a simple command line script: grep ‘⁁@’-v map.sam | cut -f3 | sort | uniq -c>result.txt. The count tables were imported to R processed using the default DESeq2 workflow,^20^ and visualized using ggplot2. Principal component analysis (PCA) of overall sample similarity was carried out using DESeq2 normalized counts (square root transformed), through the vegan^21^ ampvis R packages.^22^

KEGG pathway analysis was carried out by annotating each gene with a KO identifier using the bidirectional best hit method implemented in the KEGG Automatic Annotation Server (http://www.genome.jp/tools/kaas/).^23^ The output KO identifiers were further analyzed using MinPath,^24^ and the results were imported to R.

### Analysis of gene expression

The DESeq2 workflow was applied to normalize the read counts and identify differential expressed genes.^20^ Counts from rRNA genes were removed before the analysis. Functional enrichment analysis with regard to gene ontology categories was performed using the Cytoscape plugin BINGO.^25^ The significantly regulated pathways were selected based on the false discovery rate (Benjamini–Hochberg multiple testing correction).^26^ KEGG database was used to further analysis differentially expressed genes.^27^ Only genes which were regulated with at least twofold compared with control conditions were included for KEGG and functional enrichment analyses.

## Results

### Phenotypic effect of sertraline on tetracycline-resistant *E*.*coli*

MIC for sertraline ranged from 16 to 32 mg l^−1^ and from 32 to 1024 mg l^−1^  for tetracycline for a collection of 84 tetracycline-resistant *E. coli* isolates (Table 1). For all strains, the MIC of tetracycline could be reduced with at least 50% in the presence of ½ MIC of sertraline. For all isolates, MIC of sertraline was equivalent to MBC of the same drug.

The MICs of penicillin, erythromycin and kanamycin of *E. coli* APEC_O2 were not decreased when dilutions tubes were supplemented with ½ MIC concentrations of sertraline (Table 2), indicating that the effect was specific to tetracycline among these drugs.

**Figure 1:**
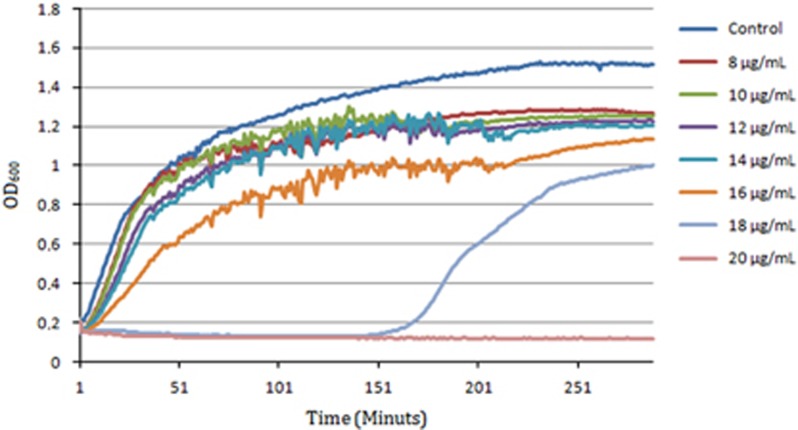
Growth curves of *E. coli* APEC_O2 exposed to increasing concentrations of sertraline.

Exposure of *E. coli* APEC_O2 to increasing concentrations of sertraline revealed that sertraline affects bacterial growth rate in a concentration-dependent manner (Figure 1), and at a concentration above ½ MIC (18 mg l^−1^), there was an increased lag phase.

Synergy as defined by Odd,^17^ corresponding to an FIC index ⩽0.5, was obtained for *E. coli* APEC_O2 with 8 mg l^−1^ sertraline and 16 mg l ^−1^ of tetracycline. The checkerboard assay also revealed that ½ MIC of sertraline (16 mg l^−1^) was sufficient to lower the MIC of tetracycline from 64 to 4 mg l^−1^.

**Figure 2:**
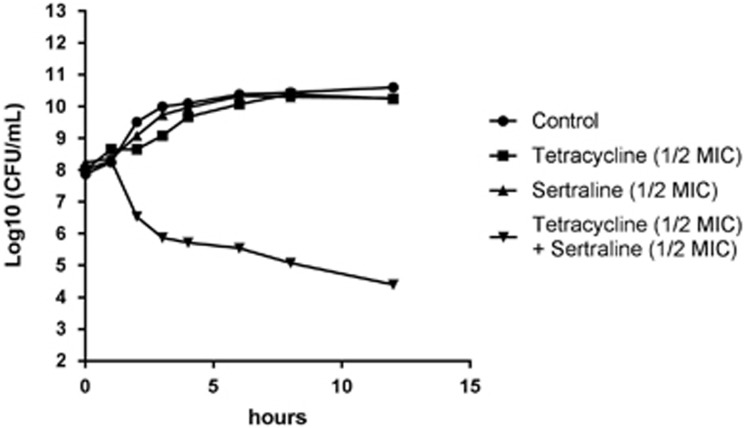
Time-kill curve of *E. coli* APEC_O2 exposed to tetracycline, sertraline, a combination thereof, or untreated control conditions (Mueller–Hinton (MH) broth).

Compound inhibition of broad-specificity efflux pumps only had limited impact on tetracycline resistance of *E*.*coli* APEC_O2 (Table 2). Time-kill curves revealed that the CFU for *E. coli* APEC_O2 exposed to ½ MIC of a combination of sertraline and tetracycline was reduced ~4 log_10_, compared with treatment with ½ MIC of sertraline, tetracycline or untreated control (Figure 2).

### Transcriptome analysis of tetracycline-resistant *E. coli* treated with sertraline

**Figure 3:**
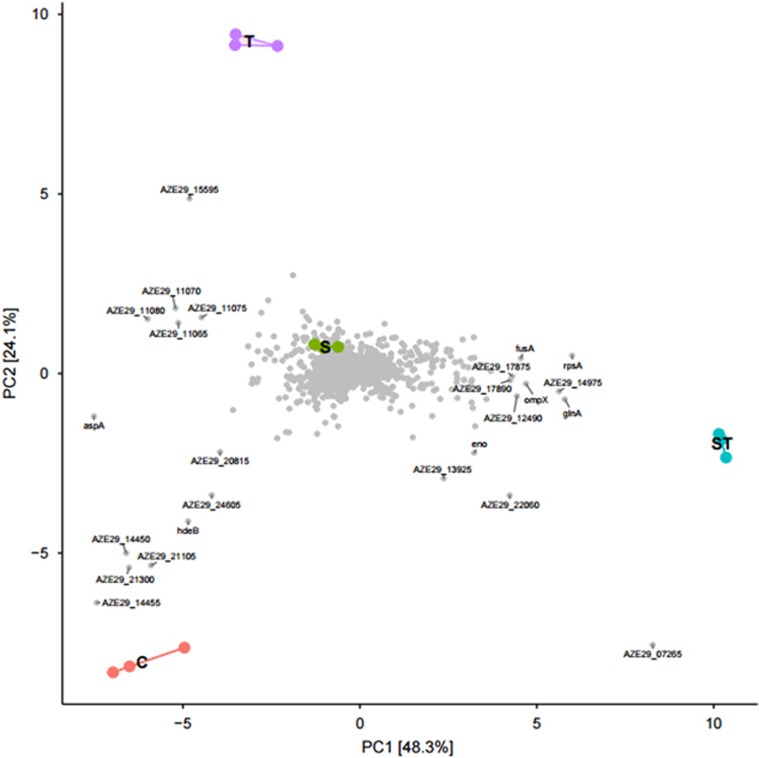
Overall sample comparison using principal component analysis (PCA). PCA was used to compare the global gene expression between all samples (C: control; S: sertraline treatment; ST: sertraline and tetracycline treatment; T: tetracycline treatment). Samples located together have similar gene expression. Genes (gray dots) located in the same direction as samples have higher expression in those sample.

**Figure 4:**
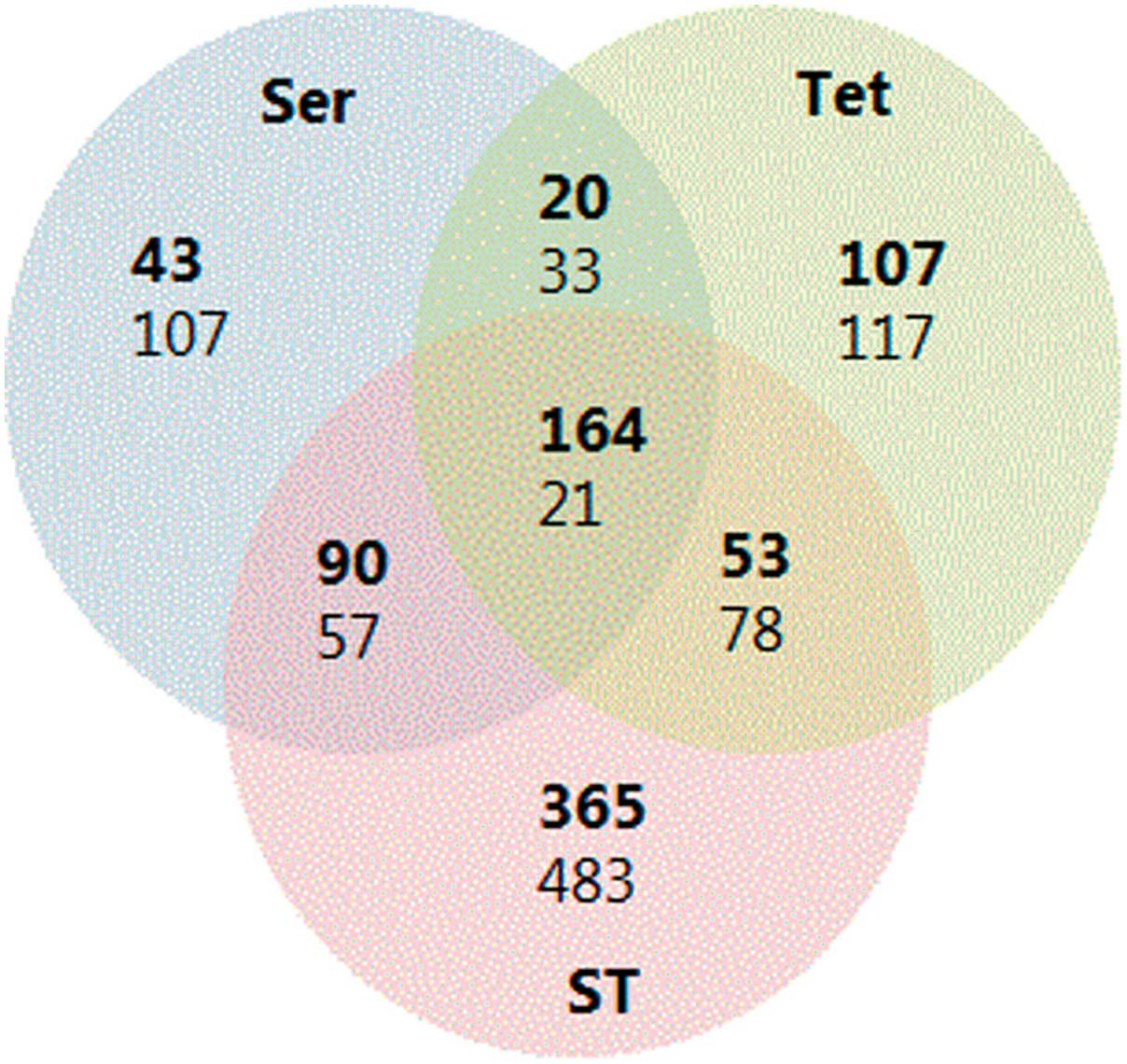
Venn diagram comparisons of control (C) vs treatments (sertraline (Ser), tetracycline (Tet), tetracycline+sertraline (ST). Numbers in bold indicate number of upregulated genes and non-bold indicate number of downregulated genes.

RNA sequencing was performed on *E*.*coli* APEC_O2 treated with tetracycline and/or sertraline. After the quality control, 1.3 to 2.2 million reads generated per sample. Of the mapped reads, ~70% mapped to annotated genes in APEC_O2. A violin plot revealed that sequencing resulted in ~50–500 reads per gene per sample, which enabled comprehensive investigation of the complete transcriptome. The analysis identified the expression of 5168 coding DNA sequencing tags. A PCA of the normalized gene expression data from DESeq2 showed that the samples tightly clustered based on treatment condition and that selected genes were upregulated under specific conditions (Figure 3). The PCA also revealed that the ST-exposed samples clustered furthest from the unexposed control on the first dimension compared (maximal variations in differentially expressed genes to sertraline- and tetracycline-treated samples). Overall, ST induced a higher number of differentially expressed genes compared with either sertraline or tetracycline treatment vs control condition (Table 3 and Figure 4).

Overviews of the 50 differentially expressed genes with lowest adjusted *P*-value (all *P*<0.001) between ST and control treatment, ST treatment vs tetracycline treatment and ST treatment vs sertraline treatment are presented in Tables 4, 5, 6, respectively. A total of 1705 significantly regulated genes could be assigned to 18 functional groups defined by KEGG (Supplementary Figure S1). Of these, 1278 were significantly regulated under ST treatment, whereas the corresponding numbers for sertraline and tetracycline treatment were 547 and 605, respectively (Table 3).

Initially, KEGG Mapper was applied to visualize pathways including genes, which were significantly regulated under ST treatment. Among the upregulated pathways were the ribosome subunits, which had 54 genes assigned, followed by the purine metabolism (21), the two-component system (20), the pyrimidine metabolism (18), carbon metabolism (13), ABC transporters (13) and glycolysis (11), while a number of other pathways had between 10 and 0 genes assigned each. Subsequently, functional enrichment analysis was applied using Cytoscape with the plugin BINGO. Here, 300 gene ontology categories were identified, of which 174 were significantly regulated (*P*<0.05). The most significantly upregulated pathways corresponded to the output from KEGG Mapper.

Expression of *tetA*, encoding the TetA pump, and *tetR*, encoding the TetA repressor, differed between *E. coli* APEC_O2 exposed to different treatments (Table 7). Under the ST and tetracycline treatments, *tet*R as well as *tet*A were significantly upregulated, the latter by more than 32-fold in both treatments, whereas neither of the genes were regulated significantly under sertraline treatment alone compared with control conditions. However, the ratio between the *tetA*:*tetR* under tetracycline treatment (2.8) was compared with the *tetA*:*tetR* ratio under ST treatment (2.4).

Genes encoding AcrAB (*acrA* and *acrB)* were the most significantly downregulated efflux pump genes across all treatment groups. Sertraline treatment induced the most significant downregulation, followed by tetracycline treatment.

Among all pathways, the ribosomal pathways had the highest number of significantly regulated genes assigned; hence, the ribosomal and purine metabolic pathways were the most significantly upregulated categories. All the ribosomal encoding genes were significantly upregulated under ST vs tetracycline treatment (Table 5) even though the tetracycline concentration was eight times in tetracycline treatment compared with ST treatment. The upregulation was not due to the effect of sertraline, as no ribosomal encoding genes were upregulated under sertraline treatment (Tables 5 and 6).

Genes encoding for glycolytic process were also among the most significantly upregulated in ST treatment vs tetracycline and sertraline treatment, and control conditions (Tables 4, 5, 6). The *citT* and *asr* genes, encoding a citrate/succinate antiporter and acid-shock protein, respectively, were more than 250-fold upregulated in ST treatment (Tables 5 and 6).

Treatment of *E. coli* APEC_O2 with ST led to 438 uniquely downregulated genes (Figure 4). KEGG Mapper revealed that these genes were involved in carbon metabolism (27), amino acids biosynthesis (21), ABC transporters (17), glyoxylate metabolism (14), starch and sucrose metabolism (11), tricarboxylic acid cycle (10), while a number of other downregulated pathways had between 9 and 0 genes assigned

In the functional enrichment analysis, the significantly downregulated genes were assigned to 120 gene ontology categories, in which 59 categories were significantly regulated. The functional analysis confirmed the findings by KEGG, and also revealed an overall decreased oxidative–reduction process pathway (*P*<0.0001). Similarly, energy derivation by oxidation of organic compounds was also highly significantly downregulated under ST treatment only (*P*<0.001).

In addition to the above, cellular respiration as well as the respiratory electron transport chain and generation of precursor metabolites pathways were among the most significantly downregulated categories.

## Discussion

In the current study, we document that sertraline in subinhibitory concentrations reduced MIC of tetracycline for 84 tetracycline-resistant strains of *E*.*coli*, and restored the sensitivity of tetracycline in *E*.*coli* APEC_O2 as defined by EUCAST.^28^ Growth curves of *E*.*coli* APEC_O2 exposed with various concentration of sertraline and/or tetracycline indicated a concentration-dependent decrease of growth rates (Figure 1), whereas the checkerboard analysis documented that the two compounds do indeed have a synergistic interaction. Combination of sertraline with other classes of antibiotics did not decrease the MIC for these antibiotics (Table 2), which also support that sertraline interacts synergistically with tetracycline, rather than considering growth inhibition as a simple ‘two-hit model’.

The synergistic interaction between sertraline and tetracycline did not occur as a result of sertraline acting as a broad-spectrum efflux inhibitor. General efflux inhibitors did not restore tetracycline sensitivity of *E*.*coli* APEC_O2, although sertraline treatment did result in reduced expression of *acr*A and *acr*B (Tables 2 and 7). In terms of antibiotic combination treatments, it is well known that bacteriostatic–bactericidal combination treatments results in attenuation of bactericidal activity *in vitro*.^29, 30, 31^ Single treatment with sertraline revealed that for this compound, MBC equaled MIC (Tables 1 and 2), a phenotypic observation indicative of a bactericidal compound.^32^ Newer research suggests that bactericidal and bacteriostatic antibiotics differentially perturb bacterial cellular respiration, in which the former accelerates respiration, whereas the latter decelerates cellular respiration.^33^ In accordance with that research, we found that tetracycline downregulated genes involved in cellular respiration, whereas sertraline treatment did not affect cellular-respiration genes. This differential activity, according to Lobritz *et al.*,^33^ would imply that sertraline is not a ‘true’ bactericidal antibiotic. However, the combined treatment of *E. coli* with sertraline and tetracycline significantly downregulated the cellular-respiration and tricarboxylic acid cycle.

**Figure 5:**
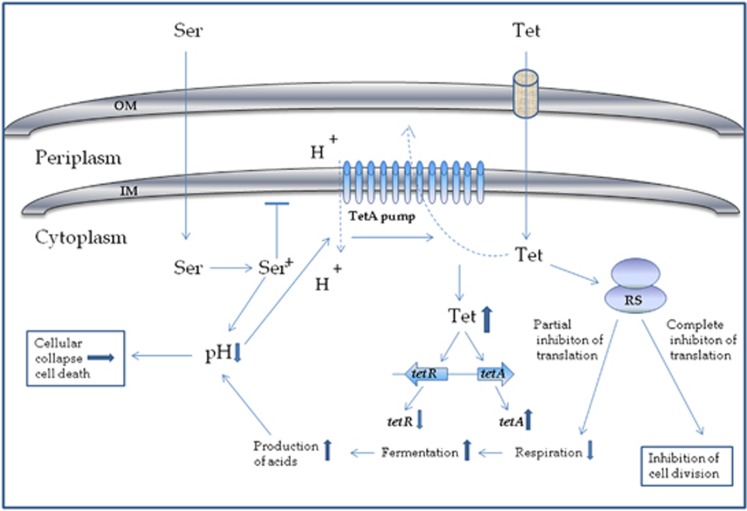
Schematic model of synergy between sertraline and tetracycline in a TetA containing *E. coli*. The model is based on RNA sequencing data and phenotypic obervations on MIC and MBC of tetracycline and sertraline, when *E. coli* APEC_O2 is exposed to each compound individually and combined. In the non-protonated form, sertraline (Ser) can pass the outer membrane (OM) and inner membrane (IM) due to the lypophilic nature of the compound. In the cytoplasm, sertraline is protonated, which causes a decrease in intracellular pH. According to the model of Berens and Hillen,^8^ tetracycline (Tet) in complex with magnesium (not shown in figure) enters the OM through porins. In the periplasm, tetracycline can dissociate from the tetracycline–magnesium complex and enter the cytoplasm by diffusion over the IM. After entering the cytoplasm, tetracycline can reach its ribomal (RS) target, unless it is actively extruted from the cell by the TetA pump, which is indicated by the 12-α-helix structur. The TetA pump is driven by a proton-motive force energy. If sertraline decreases the proton gradient, protons are not transfering through the inner membrane (indicated by a dashed line). If the TetA pump is not functional, the tetracycline concentration will increase in the cell, which leads to increasing the expression of *tetA* and decreasing concentrations of *tetR*.^13^ When the tetracycline concentration equals MIC for the strain, the translation from the ribosomes will be completely blocked and the cell will not be dividing. If tetracycline is removed, the cell will start dividing again, hence MBC is higher than MIC. In concentrations of tetracycline below MIC, the cell will continue to divide in the presence of tetracycline, yet there will be a partial shift from oxidation to fermentation. Fermenation will result in production of mixed fatty acid, which will add to a futher decrease of intracelluar pH, finally resulting in a cellular collapse and cell death. Hence, when sertraline and tetracycline are combined, the MIC of the combined exposure equals MBC. If *E. coli* is exposed only to sertraline, the cell will die when the concentrations of sertraline is high enough to cause a lethal drop in intracelluar pH. This mode of action is largely independent of strain chracteristics, and equals ~32 μg ml^−1^ for *E. coli* in general.

Respiration is necessary for the reoxidation of NADH, which is coupled to the generation of a proton gradient that is used, among many processes, to drive ATP production via proton influx through the F_1_F_0_ ATPase stator.^34^ Lack of cellular respiration will therefore be followed by a lower proton gradient, which might be further decreased by protonation of the amino group of sertraline in the bacterial cytoplasm. Acid-shock genes were more than 300 times upregulated in both ST treatment and sertraline treatment (Table 4), but downregulated under tetracycline treatment, supporting the hypothesis that sertraline significantly decreases pH. Also, *sdaC* encoding an H^+^/serine symporter^35^ were more than 20-fold downregulated in ST treatment, indicating that the bacteria is highly avoiding further acidification. It is noteworthy that, even at the MIC for sertraline (32 mg l^−1^), a decrease in pH of the growth medium by more than 0.2 was not observed, leading to the conclusion that the acid-shock response is due to *intracellular* decrease in pH only. Decrease in the proton gradient will interfere with the TetA pump, which transports tetracycline from the bacterial cytoplasm to the periplasm; the flow of protons from the periplasm to the cytoplasm provides the energy to the pump. The flow is dependent on a proton gradient between the periplasm and the cytoplasm (Figure 5).^8^

Regulation of *tetA*, the TetA pump encoding gene, and *tetR*, a TetA repressor gene, is highly dependent on the intracellular tetracycline concentration, and the ratio of expression of *tetA/tetR* increases with increasing concentration of tetracycline.^13^ Nevertheless, the results obtained in the present study revealed a similar *tetA/tetR* ratio when 32 mg l^−1^ tetracycline was administered (under tetracycline treatment) and when only 4 mg l^−1^ was administered (under ST treatment) (Table 7). If the proton gradient between the cyto- and periplasm decreases, a lower efficiency of the TetA pump will follow, and thereby a higher cytoplasmic concentration of tetracycline will be present. If so, the cytoplasmic concentration of tetracycline will be higher if sertraline is coadministered, and hence, the *tet*A/*tetR* ration would diverge from what would be expected if tetracycline in the same concentrations as used in the present study were administered without sertraline.

Decrease of the electron transport chain, as indicated by downregulation of genes (*nrfA*, *nrfD*, *nrfZ*, *cydA*, *cydB*), will require other processes to act as electron acceptors; for example, through fermentation, for the bacteria to grow. In fermentation, however, the pyruvate made in glycolysis does not continue through oxidation and the tricarboxylic acid. Essential genes in the glycolytic pathway (*pgi*, *pkg*, *pts*,*eno*, *pykA*) to generate pyruvate were all upregulated under ST treatment, as were pyruvate kinase (*pykF*), lactate dehydrogenase (*ldhA*) and alcoholdehydrogenase (*adhE*), indicating that a direct fermentation of pyruvate is occurring, generating lactate, as well as in direct conversion of pyruvate to ethanol by using acetyl-CoA as an intermediate treatment. The conversion of acetyl-CoA to ethanol is particularly important to *E*.*coli* as this reaction generates two molecules of the highly needed NAD^+^. Also in support of increased fermentation, ST treatment led to the upregulation of *mdh*, *fum* and *frd* genes, which encodes catalyzing proteins needed in the three-step conversion of phosphoenolpyruvate to succinate under fermentation, a process that also generates two molecules of NAD^+^. Genes encoding conversion of acetyl-coA to acetate (*pta*, *ackA*) were also more highly expressed under ST treatment compared with any of the other conditions. Generation of lactate and acetate will further decrease intracellular pH, which could explain why acid-shock proteins were equally upregulated during ST and sertraline treatments, although two times higher concentrations of sertraline was used in the latter treatment.

Genes encoding for citrate lyase (*citF*) and a citrate/succinate antitransporter, *citT*, were both upregulated more than 50-fold. *citF* catalyses the cleavage of citrate under anaerobic conditions, whereas *citT* is a citrate/succinate antiporter, responsible for the uptake of citrate. Under anaerobic conditions, citrate can be used if an oxidizable cosubstrate is present, such as glycerol or glucose.^36^ As succinate is also the end product of citrate fermentation, *citT* continues to feed the cell with citrate in the exchange for succinate, which is pumped out of the cell.^37^ Finally, *hyc* encoding formate hydrogenase lysase was upregulated in ST treatment only. Formate hydrogenlyase uses protons to reduce formate during mixed-acid fermentation,^38^ protecting the cell from the fermentation-related acidic conditions. These findings all support the hypothesis that fermentation is occurring under ST treatment only.

In conclusion, *in vitro* syngery between sertraline and tetracycline was observed, independently of AcrAB efflux pump inhibition. An intracellular acidification, a shift from oxidation to fermentation and decrease in PMF is suggested to be the main cause of synergy between sertraline and tetracycline in *E*.*coli* APEC_O2.

## Figures and Tables

**Table 1:** MIC values for sertraline and tetracycline for a collection of 84 tetracycline-resistant strains of *E. coli*

	*MIC*_*50*_	*MIC*_*90*_	*MIC (lowest–highest)*
Sertraline	32	32	16–32
Tetracycline	128	512	32–1024
Tetracycline (+½ MIC sertraline)	16	64	1–512

**Table 2:** MIC values for sertraline, tetracycline and tetracycline in combination with sertraline or other agents known to be inhibitors of efflux pumps in *E. coli*

	*E. coli APEC_O2*	
	*MIC (μg ml*^−1^)	*MBC (μg ml*^−1^)
Tetracycline Sertraline	64	128
	32	32
Tetracycline +1/2 MIC sertraline	4	4
Tetracycline +20 μg ml^−1^ PAN	64	—
Tetracycline+1/2 MIC TDZ	32	—
Tetracycline+1/2 MIC CHL	64	—

Abbreviations: CHL, chlorpromazine; PAN, Phe-Arg β-naphthylamide; TDZ, thioridazine.

**Table 3:** Summary of the total number of genes significantly up/down genes in *E. coli* ^a^

*Conditions compared*	*Total number of regulated genes*	*Up*	*Down*
Control vs sertraline	535	317	218
Control vs tetracycline	593	344	249
Control vs sertraline+ tetracycline	1311	672	639
Sertraline vs sertraline+tetracycline	1002	530	472
Tetracycline vs sertraline+tetracycline	1138	601	537
Sertraline vs tetracycline	640	324	316

Abbreviations: Up/down, up- or downregulated.

a APEC_O2 exposed to sertraline, tetracycline or a combination hereof vs untreated control conditions.

**Table 4:** The 50 most significant differentially expressed genes in *E. coli* ^a^

*Relative expressions of mRNA*	*Description (functional group)*	*Treatment*		
		*Ser+Tet (8 mg l*^−1^*+4 mg l*^−1^)	*Tet (32 mg l*^−1^)	*Ser (16 mg l*^−1^)
*Downregulated*				
*ridA*	Reactive intermediate/imine deaminase (V)	**−5.57**	0.05	**0.90**
*yghZ*	Aldo/keto reductase (−)	**−3.41**	0.14	−0.10
*osmY*	Hypothetical protein (S)	**−4.77**	**0.53**	0.11
*malP*	Maltodextrin phosphorylase (G)	**−4.11**	**−1.08**	**−2.58**
*elaB*	Protein ElaB (J)	**−4.15**	**−1.58**	**−1.27**
*dhnA*	Fructose-bisphosphate aldolase (G)	**−5.13**	**−3.12**	**−0.73**
*lacZ*	Beta-d-galactosidase (G)	**−6.69**	0.17	**−0.55**
*celF*	Alpha-glucosidase/alpha-galactosidase (G)	**−5.53**	**0.13**	**−0.63**
*malQ*	4-alpha-glucanotransferase (G)	**−4.77**	**−0.92**	**−2.29**
NA	Hypothetical protein (−)	**−5.97**	**−5.38**	**−4.86**
*brf*	Bacterioferritin (P)	**−4.46**	**−2.11**	**−1.73**
*pduW*	Propionate kinase (C)	**−6.40**	0.38	**1.16**
*hchA*	Chaperone protein HchA (R)	**−4.44**	**−2.02**	**−1.80**
*ppsA*	Phosphoenolpyruvate synthase (G)	**−3.79**	**−0.41**	**0.57**
NA	Hypothetical protein (−)	**−5.00**	**−0.98**	**−2.01**
*wrbA*	NAD(P)H:quinone oxidoreductase (C)	**−5.00**	−0.90	**−1.98**
*lamB*	Maltoporin (G)	**−6.92**	**−1.05**	**−6.91**
*ilvA*	Threonine dehydratase (E)	**−6.01**	0.22	**−0.92**
*tdcE*	PFL-like enzyme TdcE (C)	**−5.55**	−0.08	**1.22**
*sdaC*	HAAAP family serine/threonine permease (E)	**−5.93**	0.45	**1.55**
*gadA*	Glutamate decarboxylase (E)	**−5.43**	**−5.19**	**−3.48**
*malE*	Maltose ABC transporter substrate-binding protein (G)	**−6.64**	**−2.02**	**−5.40**
*gadC*	Glutamate:gamma-aminobutyrate antiporter	**−5.71**	**−4.82**	−3.56
*flgL*	Flagellin (N)	**−6.93**	**−5.66**	**−6.98**
NA	Hypothetical protein (−)	**−2.77**	**−0.88**	**−0.96**
				
*Upregulated*				
*rpo*A	DNA-directed RNA polymerase subunit alpha (K)	**2.13**	**0.77**	0.06
*rpl*O	50S ribosomal protein L15 (J)	**2.24**	**0.84**	0.27
*rpl*J	50S ribosomal protein L10 (J)	**2.23**	**0.45**	0.28
*gln*H	Glutamine ABC transporter substrate-binding protein (E)	**2.53**	−0.82	−0.22
*rpp*L	50S ribosomal protein L16 (J)	**2.50**	0.32	0.18
*mdh*	Malate dehydrogenase (C)	**2.15**	**0.56**	**1.75**
*citF*	Citrate lyase subunit alpha (C)	**6.63**	**−1.25**	**5.58**
*typ*A	GTP-binding protein TypA (T)	**2.60**	0.38	**0.63**
*rpl*K	50S ribosomal protein L11 (J)	**2.22**	**0.45**	0.03
*pta*N	Phosphate acetyltransferase (R)	2.38	**−1.35**	**1.18**
*gln*Q	Glutamine ABC transporter ATP-binding protein (E)	**3.23**	−0.45	0.19
*pri*B	Primosomal replication protein N (L)	**2.69**	**0.82**	**0.47**
*rpl*Q	50S ribosomal protein L17 (J)	**2.18**	**0.97**	0.08
*rpl*A	50S ribosomal protein L1 (J)	**2.34**	**0.55**	0.09
*rps*F	30S ribosomal protein S6 (J)	**2.77**	**0.71**	**0.32**
*ack*A	Acetate kinase (C)	**2.45**	**−1.08**	**0.88**
*guaB*	IMP dehydrogenase (T)	**4.23**	**1.27**	**1.37**
*glnA*	Glutamine synthetase (E)	**4.25**	−0.19	**0.82**
*pyk*F	Pyruvate kinase (G)	**2.61**	**−0.73**	−0.21
*alk*P	2,3-bisphosphoglycerate-independent phosphoglycerate mutase (G)	3.02	**−0.67**	0.09
*pts*G	PTS glucose-specific subunit IIBC (G)	**3.57**	−0.28	0.05
*gua*A	GMP synthase (E)	**3.49**	0.18	**0.80**
*cit*T	Anion permease (G)	**8.31**	−0.76	**6.49**
*asr*	Acid-shock protein (−)	**8.29**	**−1.31**	**8.97**

Abbreviations: Ser, sertraline; Tet, tetracycline.

a APEC_O2 treated with either Ser or Tet combined Tet and Ser (ST) vs control conditions. The change in expression is relative to control condition, and values are stated as log 2 fold-change. For completeness, the gene regulatory levels of Ser- and Tet-only treatments vs control condition for the same 50 genes are also included in the table. Values in bold are significantly different from control.

**Table 5:** The 50 most significant differential expressed genes in *E. coli* ^a^

*Relative expressions of mRNA*	*Description (functional group)*^b^	*Treatment*		
		*Ser+Tet (8 μg ml*^−1^*+4 μg ml*^−1^)	*Tet (32 μg ml*^−1^)	*Ser (16 μg ml*^−1^)
*Upregulated*				
*pkg*	Phosphoglycerate kinase (G)	**1.79**	**−0.53**	**−0.33**
*pgi*	Glucose-6-phosphate isomerase (G)	**0.73**	**−1.16**	−0.25
*aph*C	Alkyl hydroperoxide reductase (V)	**1.36**	**−0.97**	0.08
*foc*A	Formate transporter (P)	**0.75**	**−1.96**	0.25
*mnh*D	Formate hydrogenlyase subunit (C)	**1.59**	**−3.09**	**−0.69**
*pot*E	Arginine:agmatine antiporter (E)	**1.53**	**−3.42**	**−3.37**
*tuf*	Elongation factor Tu (−)	**1.55**	0.12	**0.55**
*sdh*A	Fumarate reductase (C)	**0.75**	**−1.29**	**0.73**
*hyc*E	Hydrogenase 3 large subunit (C)	**1.59**	**−2.53**	**−0.30**
NA	Transcriptional regulator (−)	**1.77**	**−3.01**	**−1.01**
*pts*H	PTS sugar transporter (G)	**2.22**	**−0.71**	**0.81**
*gln*Q	Glutamine ABC transporter ATP-binding protein (E)	**3.32**	**−3.38**	**0.20**
*nrd*D	Anaerobic ribonucleoside triphosphate reductase (F)	**2.24**	**−0.80**	**1.07**
*sse*A	Thiosulfate sulfurtransferase (P)	**1.65**	**−2.14**	**0.41**
*tpi*A	Triose-phosphate isomerase (G)	**1.00**	**−1.56**	**0.38**
*gln*A	Glutamine synthetase (E)	**4.25**	−0.19	**0.80**
*gln*H	Glutamine ABC transporter substrate-binding protein (E)	**2.53**	**−0.83**	**−0.22**
*pts*A	Phosphoenolpyruvate-protein phosphotransferase (G)	**1.54**	**−3.22**	**0.17**
*gua*A	GMP synthase (E)	**3.50**	0.18	**0.81**
*idc*C	Arginine decarboxylase (E)	**1.72**	**−3.05**	**−3.20**
*asr*	Acid-shock protein (−)	**8.29**	**−1.31**	**8.97**
*potA*	Spermidine/putrescine ABC transporter substrate-binding protein (−)	**1.67**	**−2.01**	0.02
*fdhF*	Formate dehydrogenase subunit alpha	**1.56**	**−1.09**	0.28
*pts*G	PTS glucose-specific subunit IIBC (G)	**3.57**	−2.28	0.01
*cit*T	Anion permease (G)	**8.31**	**−2.27**	**6.49**
*eno*	Enolase (G)	**1.25**	**−1.59**	**0.12**
*alk*P	2,3-bisphosphoglycerate-independent phosphoglycerate mutase (G)	**3.01**	**−0.67**	**0.08**
*pta*N	Phosphate acetyltransferase (R)	**2.38**	**−1.35**	**1.18**
*pyk*F	Pyruvate kinase (G)	**2.61**	**−0.73**	−0.20
*ack*A	Acetate kinase (C)	**2.45**	**−1.08**	**0.88**
*gap*A	Glyceraldehyde-3-phosphate (G)	**1.22**	**−1.85**	**0.19**
*fab*	Class II fructose-bisphosphate aldolase (G)	**0.91**	**−1.83**	**−0.20**
*pfl*D	Pyruvate formate-lyase (C)	**1.24**	**−2.82**	**0.05**
				
*Downregulated*				
*yghZ*	Oxidoreductase (−)	**−3.41**	0.14	0.10
*ridA*	Reactive intermediate/imine deaminase (V)	**−5.57**	0.05	**0.90**
*traT*	Conjugal transfer surface exclusion protein TraT (−)	**−0.80**	**1.55**	**0.63**
NA	Hypothetical protein (−)	**−5.00**	**−0.99**	**−2.08**
*wrbA*	NAD(P)H:quinone oxidoreductase (C)	**−5.00**	**−0.90**	**−1.98**
*glgS*	Glycogen synthesis protein (−)	**−2.25**	**2.16**	−0.29
*traS*	Conjugal transfer protein TraS	−0.12	**1.82**	**0.28**
*malE*	Maltose ABC transporter substrate-binding protein MalE (G)	**−6.64**	**−2.02**	**−5.40**
*lamB*	Maltoporin (G)	**−6.72**	**−1.05**	**−6.19**
*lacZ*	Beta-d-galactosidase (G)	**−6.89**	0.17	**−0.65**
*celF*	Alpha-glucosidase/alpha-galactosidase (G)	**−5.53**	0.13	**0.63**
*ppsA*	Phosphoenolpyruvate synthase (G)	**−3.79**	**−0.41**	**0.57**
*tdcD*	Propionate kinase (C)	**−6.40**	0.38	**1.16**
*tdcE*	PFL-like enzyme TdcE (C)	**−5.54**	0.08	**1.23**
*ilvA*	Threonine dehydratase (E)	**−6.08**	**0.86**	**0.01**
*sdaC*	HAAAP family serine/threonine permease	**−5.93**	−0.45	**1.15**

Abbreviations: Ser, sertraline; Tet, tetracycline.

a APEC_O2 treated with Ser or Tet or combined Ser and Tet vs single treatment with Tet. The change in expression is relative to control condition, and values are stated as log 2 fold-change. For completeness, the gene regulatory level of Ser vs control condition for the same 50 genes is also included in the table. Values in bold are significantly different from control.

b According to KEGG classification: C, energy production and conversion; E, amino-acid transport and metabolism; V, defense mechanism; G, carbohydrate transport and metabolism; P, inorganic transport and metabolism; T, signal-transduction mechanisms; J, translation, ribosomal structure and biogenesis; R, general function prediction only; NA, not available.

**Table 6:** The 50 most significant differential expressed genes in *E. coli* ^a^

*Relative expression of mRNA*	*Description (functional group)*^b^	*Treatment*		
		*Ser+ Tet (8 μg ml*^−1^*+4 μg ml*^−1^)	*Tet (32 μg ml*^−1^)	*Ser (16 μg ml*^−1^)
*Upregulated*				
*pykF*	Pyruvate kinase (G)	**2.62**	**−0.73**	−0.20
*ptsG1*	PTS glucose-specific subunit IIBC (G)	**3.57**	−0.28	0.01
*ldcC*	Arginine decarboxylase	**1.72**	**−3.05**	**−3.20**
*alkP*	Phosphoglycerate mutase (G)	**3.02**	**−0.67**	0.08
NA	Hypothetical protein (S)	−0.19	**−0.92**	**−4.25**
*rplA*	50S ribosomal protein L1 (J)	**2.34**	**0.55**	0.09
*glnH*	Glutamine ABC transporter substrate-protein (C)	**2.54**	**−0.83**	−0.23
*guaA*	GMP synthase (E)	**3.50**	0.18	**0.81**
*rplQ*	50S ribosomal protein L17 (J)	**2.18**	**0.97**	0.08
*rplK*	50S ribosomal protein L11 (J)	**2.22**	**0.45**	−0.03
*rpsF*	30S ribosomal protein S6 (J)	**2.77**	**0.71**	0.32
*glnA*	Glutamine synthetase (E)	**4.25**	−0.19	**0.82**
*glnQ*	Glutamine ATP-binding protein (E)	**3.23**	−0.45	0.19
*rpoA*	DNA-directed RNA polymerase subunit alpha (K)	**2.13**	**0.78**	0.05
*rpsQ*	30S ribosomal protein S17 (J)	**2.16**	**0.43**	0.02
*rpsD*	30S ribosomal protein S4 (J)	**1.97**	**0.64**	0.00
*rppL*	50S ribosomal protein L16 (J)	**2.50**	0.32	0.18
*potE*	Arginine:agmatine antiporter (E)	**1.53**	**−3.25**	**−3.74**
*hisJ*	Amino-acid transporter (E)	**2.04**	−0.44	**−0.80**
NA	50S ribosomal protein L29 (J)	**2.56**	**0.47**	0.18
*araC*	Transcriptional regulator (K)	**2.25**	**−1.20**	**−2.15**
*rpsM*	30S ribosomal protein S13 (J)	**1.95**	**0.54**	−0.03
*pgk*	Phosphoglycerate kinase (G)	**1.80**	**−0.54**	−0.32
*rplJ*	50S ribosomal protein L10 (J)	**2.33**	**0.45**	0.28
*rpsK*	30S ribosomal protein S11 (J)	**2.05**	**0.74**	0.04
*priB*	Primosomal replication protein N (L)	**2.69**	**0.82**	**0.47**
				
*Downregulated*				
*sdaC*	HAAAP family serine/threonine permease (E)	**−5.94**	0.45	**1.15**
*tdcE*	PFL-like enzyme TdcE (C)	**−5.54**	0.08	**1.22**
*ilvA*	Threonine dehydratase (E)	**−6.08**	0.21	**0.91**
*rbsB*	ABC transporter substrate-binding protein (G)	**−2.64**	0.28	**3.10**
*ackA*	Propionate kinase (C)	**−6.41**	0.38	**1.16**
*ppsA*	Phosphoenolpyruvate synthase (G)	**−3.79**	**−0.41**	**0.57**
*galT*	Galactokinase (G)	**−2.19**	**0.92**	**1.75**
*galM*	Galactose mutarotase (G)	**−1.94**	**0.72**	**1.77**
*ridA*	Reactive intermediate/imine deaminase (−)	**−5.58**	−0.05	**0.90**
*csrA*	Carbon starvation protein A (−)	**−2.57**	**−0.72**	**2.09**
*glpQ*	Glycerophosphoryl diester phosphodiesterase (I)	−0.53	**0.87**	**3.26**
*lacZ*	Beta-d-galactosidase (G)	**−6.70**	0.17	**−0.55**
*nuoG*	Oxidoreductase (−)	**−3.41**	−0.14	−0.10
*celF*	Alpha-glucosidase/alpha-galactosidase (G)	**−5.53**	0.13	**−0.62**
*ansB*	l-asparaginase (J)	**−2.07**	**0.98**	**1.28**
*rbsK*	Ribokinase (G)	**−0.66**	0.14	**2.55**
*sdaA*	Serine dehydratase (E)	**−5.45**	−0.04	**1.24**
*dhnA*	Fructose-bisphosphate aldolase (G)	**−5.13**	**−3.12**	**−0.72**
*galE*	Galactose-1-phosphate uridylyltransferase (G)	**−1.64**	**0.66**	**1.43**
*proP*	d-galactose transporter GalP (−)	**−2.18**	**−0.54**	**0.43**
*rbsC*	Ribose ABC transporter permease (G)	**−1.33**	0.47	**3.45**
*glpT*	Glycerol-3-phosphate transporter (G)	**2.16**	**2.37**	**4.98**
NA	Hypothetical protein (−)	0.09	0.24	**2.42**

Abbreviations: Ser, sertraline; Tet, tetracycline.

a APEC_O2 treated with Ser or Tet or combined Ser and Tet vs individual sertraline treatment. The change in expression is relative to control condition, and values are stated as log 2 fold-change. For completeness, the gene regulatory levels of individually tetracycline treatments vs control condition for the same 50 genes are also included in the table. Values in bold are significantly different from control.

b According to KEGG classification: C, energy production and conversion; E, amino-acid transport and metabolism; V, defense mechanism; G, carbohydrate transport and metabolism; P, inorganic transport and metabolism; T, signal-transduction mechanisms; J, translation, ribosomal structure and biogenesis; R, general function prediction only; NA, not available.

**Table 7:** Regulation of *tet*R, *tet*A and genes related to porin and efflux pump regulator/transport of *E. coli* ^a^

*Relative expression of genes*	*Treatment*		
	*Sertraline*	*Tetracycline*	*Sertraline+tetracycline*
*tetA*	−0.70	6.17^b^	5.64^b^,^c^
*tetR*	−0.02	2.23^b^	2.35^b^,^d^
*ompC*	1.03^b^	2.32^b^	0.48^b^,^c^
*ompX*	0.40^b^	−0.11	1.95^b^,^c^
*ompA*	0.17	0.40^b^	0.96^b^,^c^
*ompR*	0.04	−0.35	0.64^b^,^c^
*acrA*	−3.07^b^	−2.18^b^	−0.92^b^,^c^
*acrB*	−2.80^b^	−2.56^b^	−1.01^b^,^c^
*arcR*	0.21	0.11	−0.61^b^,^c^
*tolC*	−0.13	−0.75^b^	0.12^e^
*marR*	0.87	1.15^b^	2.66^b^,^c^
*emrA*	1.28^b^	1.46^b^	2.43^b^,^c^
*emrB*	0.58	0.61	1.64^b^,^c^
*emrD*	−0.64	0.68^b^	0.37^d^
*silA*	−0.45	−0.25	−1.35^b^,^c^
*silE*	−0.71^b^	0.64^b^	0.35
*silS*	−0.37	−0.44	−1.51^b^,^c^
*silR*	−0.56	−0.56	−0.98^b^
*silC*	−0.48	0.01	−0.88^b^
*bla*_*TEM-1*_	0.12	0.33	−0.35^b^,^c^

a APEC_O2 under treatment with sertraline (16 mg l^−1^), tetracycline (32 mg ml^−1^) or sertraline/tetracycline (8 and 4 mg l^−1^, respectively), compared with untreated control. Only genes of which at least one treatment was significantly different from untreated is stated in the table. Change in regulation is stated as log 2 to value.

b Significantly different from control.

c Significant difference between sertraline/tetracycline vs control, sertraline and tetracycline, respectively.

d Sertraline/tetracycline different from sertraline treatment.

e Sertraline/tetracycline significantly different from tetracycline treatment.
