# Supplementary figures and images for: Insight into synergetic mechanisms of tetracycline and the selective serotonin reuptake inhibitor, sertraline, in a tetracycline-resistant strain of Escherichia coli
Source: J Antibiot (Tokyo). 2017 Jul 12;70(9):944–53. doi: 10.1038/ja.2017.78 (PMC5589974; doi:10.1038/ja.2017.78)

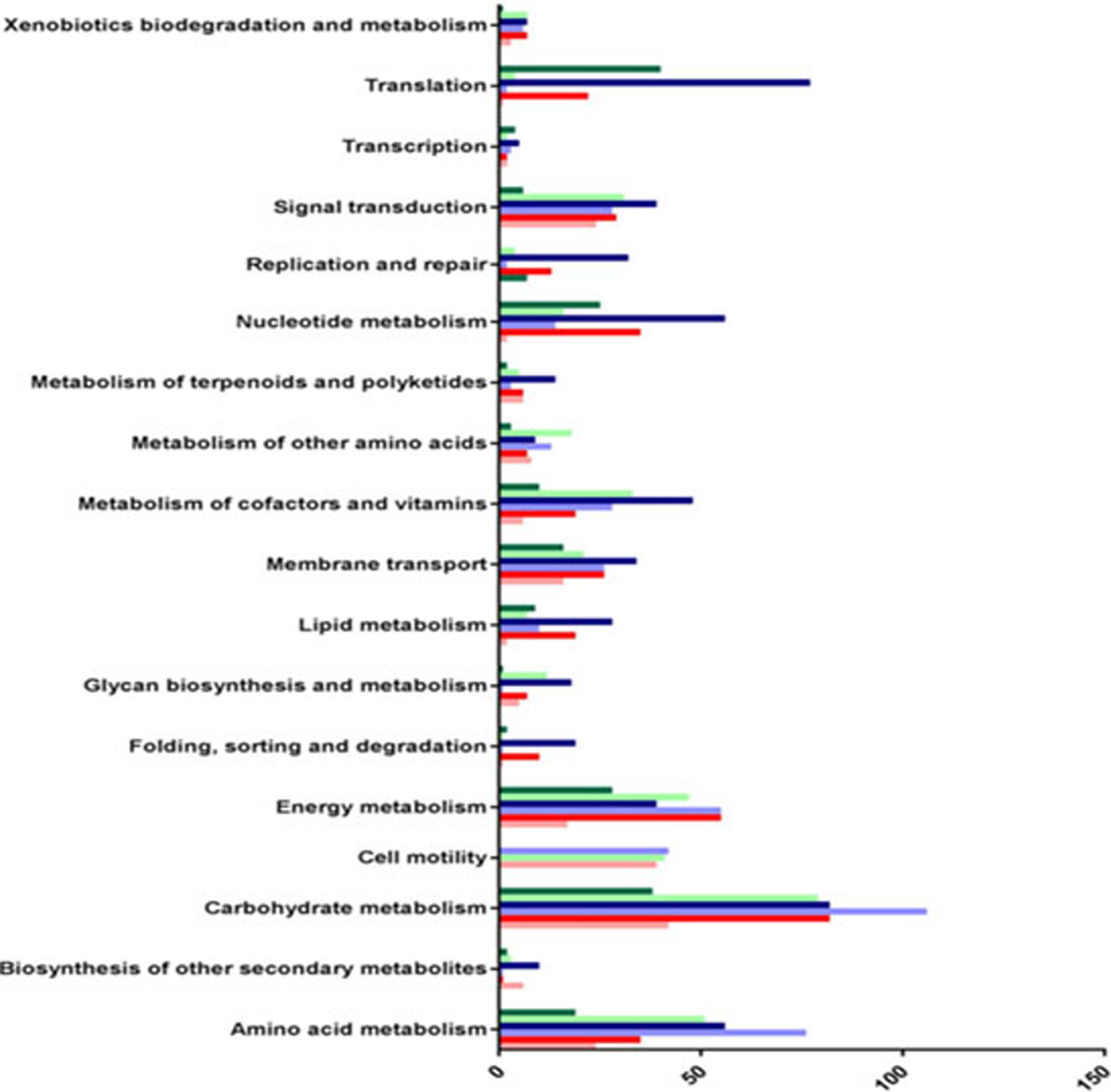

Supplement: Supplementary Figure S1 [file ja201778x1.tif]
